# Molecular detection and genetic characterization of bovine hepacivirus identified in ticks collected from cattle in Harbin, northeastern China

**DOI:** 10.3389/fvets.2023.1093898

**Published:** 2023-03-01

**Authors:** Sheng Yuan, Xin-Yan Yao, Chun-Yang Lian, Sa Kong, Jian-Wei Shao, Xue-Lian Zhang

**Affiliations:** ^1^School of Life Science and Engineering, Foshan University, Foshan, China; ^2^Beijing Biomedical Technology Center of Jofunhwa Biotechnology (Nanjing) Co., Ltd., Beijing, China

**Keywords:** bovine hepacivirus, subtype G, genetic diversity, tick, Northeastern China

## Abstract

Bovine hepacivirus (BovHepV) is a member of the genus *Hepacivirus* of the family *Flaviviridae*, which can cause acute or persistent infections in cattle. Currently, BovHepV strains identified in cattle populations worldwide can be classified into two genotypes with eight subtypes in genotype 1. BovHepV has been identified in a wide geographic area in China. Interestingly, the viral RNA of BovHepV has also been detected in ticks in Guangdong province, China. In this study, *Rhipicephalus microplus* tick samples were collected in Heilongjiang province, northeastern China, and BovHepV was screened with an overall positive rate of 10.9%. Sequence comparison and phylogenetic analysis showed that the BovHepV strains detected in this study belong to the subtype G. This is the first report about the detection of BovHepV in ticks in Heilongjiang province, China, which expands our knowledge that ticks may be a transmission vector of BovHepV.

## 1. Introduction

The genus *Hepacivirus*, which belongs to the family *Flaviviridae*, comprises a genetically diverse group of human and animal pathogens (1). The genome of the hepacivirus is about 10 kb in length, which contains the 5′ untranslated regions (UTR) and 3′ UTR, and a single large open reading frame (ORF) that encodes a single polyprotein (2). The polyprotein is cleaved by signal peptidase, NS2/NS3 protease and NS3 protease enzymes into three structural proteins (Core, E1, and E2) and seven non-structural proteins (p7, NS2, NS3, NS4A, NS4B, NS5A, and NS5B) (3, 4).

Hepaciviruses have been identified from a wide variety of mammalian hosts and non-mammalian hosts, such as bats (5), rodents (6, 7), monkeys (8), horses (9), dogs (10), donkeys (11), catshark (12), duck (13), fish and vertebrates (14–16). Currently, members of the genus *Hepacivirus* have been divided into *Hepacivirus A–N* based on their phylogenetic relationships and host range (17). In addition, more hepaciviruses have been identified in non-vertebrate hosts, such as mosquitos and ticks (18, 19), although their route of infection and transmission is uncertain.

Bovine hepacivirus (BovHepV) is the only member of the species *Hepacivirus N* and likely only infects cattle (20). It was first identified in cattle in Germany and Ghana in 2015 (21, 22). Thereafter, BovHepV has been detected in China (23–25), Brazil (26, 27), Turkey (28), the USA (29), and Italy (30), suggesting the worldwide geographic distribution of BovHepV. Moreover, BovHepV presented highly genetic diversity. As per the genotyping and subtype classification criteria for the hepatitis C virus, the BovHepV strains can be divided into two genotypes, and genotype 1 could be divided into eight subtypes (A to H) (24). Recently, BovHepV identified in Inner Mongolia, northeastern China further divided subtype G into subtypes G1 and G2, indicating the extensive genetic diversity of BovHepV (31).

In China, BovHepV have been determined in cattle herds in Guangdong, Jiangsu, Yunnan, Sichuan, Heilongjiang, Shandong, Henan, Inner Mongolia and Chongqing, with the positive rate of viral RNA ranging from 2.78 to 13.3% (25, 31–34), indicating that BovHepV was circulating in cattle herds in a wide geographic in China. Notably, the viral RNA of BovHepV has also been detected in ticks collected from cattle in Guangdong province, suggesting that tick maybe play an important role in the transmission of BovHepV among cattle (24). A previous study has shown that the prevalence of BovHepV in cattle herds in Heilongjiang province was 6.0% (25). However, no information about the epidemiology of BovHepV in ticks in Heilongjiang province is available. Therefore, in this study, blood-sucking ticks were collected from the body surface of cattle in Heilongjiang province to investigate the presence of BovHepV.

## 2. Materials and methods

### 2.1. Tick sample collection and RNA extraction

**Figure 1:**
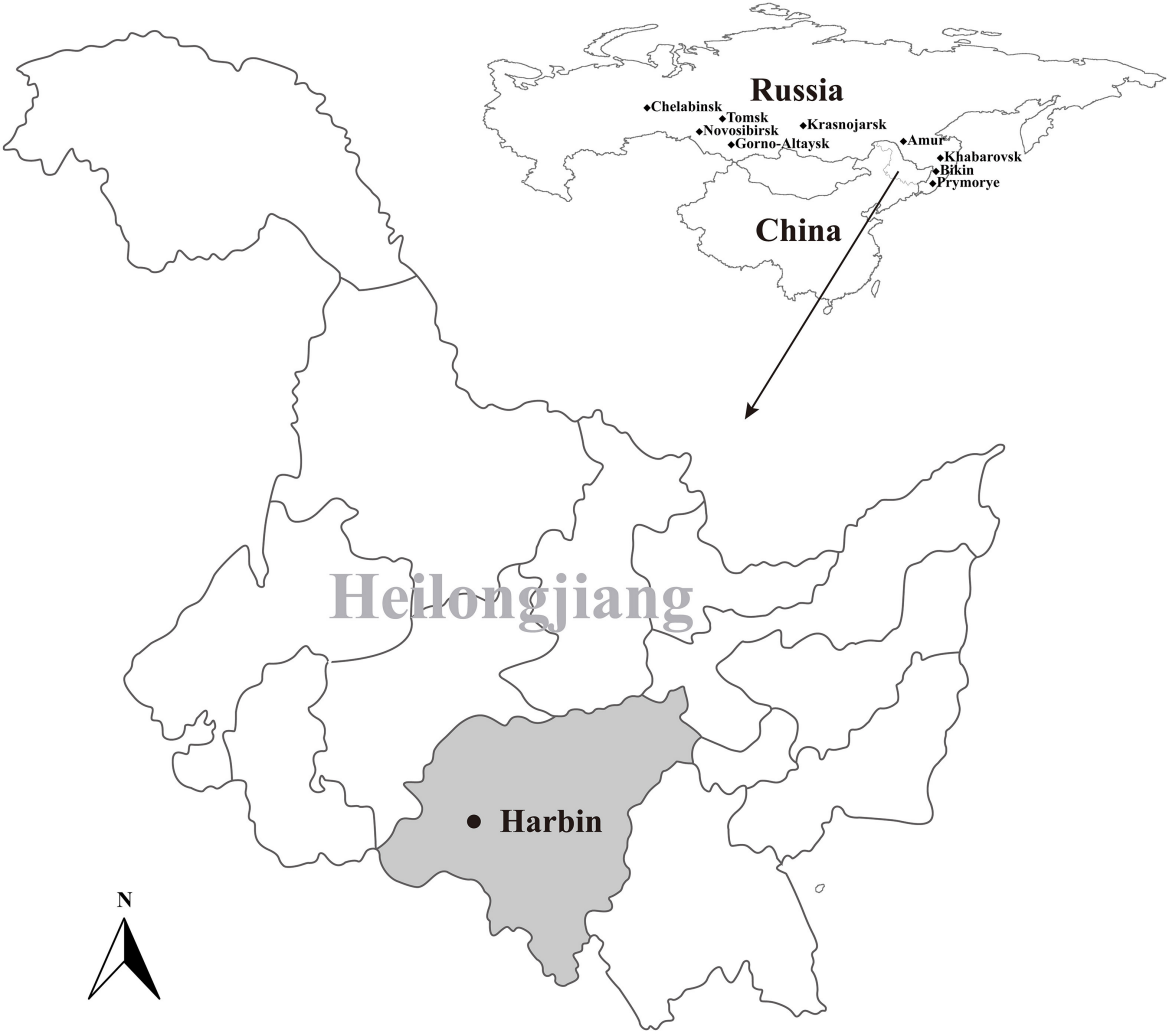
Geographic maps showing the location of sampling sites from where the rodents were captured in this study. This map was plotted using a combination of Surfer software, Version-4 (Golden Software, Golden, CO, USA), and Adobe illustrator, Version CC2017 (Adobe, San Jose, CA, USA). The black dots indicate the sampling regions in this study.

During June to August in 2021, a total of 400 blood-sucking adult tick were simple random collected from 18 cattle herds in Harbin, Heilongjiang, northeastern China (Figure 1). The tick species were identified following morphological criteria and further confirmed by sequencing and analyzing the 16S ribosomal RNA (*rrs*) gene of ticks (35). Five ticks were collected from one cattle individual and merged as one pool and stored at −80°C for further use.

Each pooled tick sample was soaked in 70% ethanol for 30 min, and washed with double distilled water three times. The samples were homogenized in 500 μL sterile phosphate buffered saline (PBS), and their total RNA was extracted from 200 μL homogenates using the TRIzol LS reagent (Invitrogen, Carlsbad, CA, USA) and subsequently purified using the RNeasy Plus Mini Kit (Qiagen, Hilden, Germany). The extracted RNA was stored at −80°C until further use.

The prevalence of BovHepV in this study was estimated assuming perfect sensitivity and specificity of molecular detection using the EPITOOLS online statistical program “Pooled prevalence for fixed pool size and perfect test” that based on the model established by Cowling et al. (36).

### 2.2. BovHepV screening and genome sequencing

Primer pairs designed based on the alignment results of the NS3 region of all BovHepV available in GenBank database were used to detect the presence of BovHepV RNA in blood-sucking ticks. Using nested RT-PCR with the primer pair BovHepV-F1/BovHepV-R1 and BovHepV-F2/BovHepV-R2, the PCR product with 493 bp was confirmed by Sanger sequencing. In addition, over-lapping primers were designed to obtain the complete genome sequence of the BovHepV identified in this study. All primers used in this study were listed in Table 1.

**Table 1:** Primers used in this study.

**Primers**	**Sequence (5^′^ → 3^′^)**	**Amplicon (bp)**	**Usage**
BovHepV-F1	GCTCARAAYCTRTGGGCKGT	981	Positive sample screening
BovHepV-R1	GARTTAGCTGGWACWGTTTTG		
BovHepV-F2	TGGGARGTYCARACYGTCTA	493	
BovHepV-R2	TTTGGAARATYARATGHCTRCC		
BovHepV-1F	ATGGAAGTYTCWGTCTCAAG	1,573	Complete genome sequence amplification
BovHepV-1572R	TCTAGCAGCAGTGGTAATGTT		
BovHepV-1693R	CCAGARTAGATRAYYTGCAT		
BovHepV-1297F	ACRTGGAGCTGYTGYAGCCT	1,568	
BovHepV-1305F	CTGYTGYAGCCTCATGGACCG		
BovHepV-2872R	GCCATRTGCATGGCRTARTC		
BovHepV-2722F	GGYAAYATYTTYACYATGGG	1,603	
BovHepV-2731F	TTYACYATGGGYACYATGTC		
BovHepV-4333R	GGRTTRCAGCCRTTCTCATA		
BovHepV-4138F	ATGTCHCCTGCDGAGGTYCT	1,612	
BovHepV-4210F	GTGCTRTCTGARATATCTGC		
BovHepV-5821R	ACRTCNGGAGARGAMGTGGC		
BovHepV-5668F	CTYTGYCGSAATTAYTGGAC	1,510	
BovHepV-5746F	TGGAAGACSATGACYGTBCA		
BovHepV-7255R	GAGTCGAADGTGATCACRTC		
BovHepV-7081F	ATAGCTTAYCCYCACCTTGA	1,220	
BovHepV-7121F	TGGTRCTYGGCAAYATAGGA		
BovHepV-8340R	TCAATGTTTGAGGAAAAAGAACAG		

The PCR products of the expected size, according to each set of primers, were purified using Gel Extraction kit (TaKaRa, Dalian, China) after electrophoresis. The purified DNA was cloned into pMD19-T vector (TaKaRa, China), and the resulting plasmid was used to transform competent *E. coli* cells. Positive inserts were confirmed by PCR, and further sequenced by Sangon Biotechnology Company (Shanghai, China). To prevent contamination, the preparation of the PCR mix and the addition of the template DNA were performed in separate rooms using dedicated pipets and filtered tips.

### 2.3. Sequence comparison and phylogenetic analysis

Sequence assembly and manually editing was performed using the SeqMan program (DNASTAR, Madison, WI), and the nucleotide (nt) and sequence identity were calculated by MegAlign program available within the Lasergene software package (version 7.1, DNAstar). Maximum-likelihood (ML) trees were reconstructed using MEGA version 7.0 (37), based on the best-fit nucleotide substitution model General Time Re-versible (GTR) nucleotide substitution model and optimized parameters of gamma (G)-distribution and proportion of invariable sites (i.e., GTR+G+I) determined by jModel Test (38). Bootstrap values were calculated from 100 replicates, and the phylogenetic trees were mid-point rooted for purposes of clarity only.

### 2.4. Recombination analysis

The seven methods (RDP, GENECONV, bootscan, maximum chi square, Chimera, SISCAN, and distance plot) within RDP4 program (39) were used to determine the potential recombination events that occurred in the evolutionary history of BovHepV. The analyses were performed based on the complete genome sequences with default settings for the different test methods and a Bonferroni corrected *p*-value cutoff of 0.05. Only sequences with significant evidence (*p* < 0.05) of recombination detected by at least two methods and confirmed by phylogenetic analysis were taken to represent strong evidence for recombination. Additionally, similarity plot analyses were inferred to further characterize potential recombination events, including the location of possible breakpoints, as implemented in Simplot version 3.5.1 (40).

### 2.5. Ethics statement

The study involving animals were reviewed and approved by the ethics committee of College of Life Science and Engineering, Foshan University. Written informed consent was obtained from the owners for the participation of their animals in this study.

## 3. Results

### 3.1. Detection of BovHepV in tick sample

A total of 400 ticks, which were identified as *Rhipicephalus microplus*, were collected from 18 cattle herds in Harbin, Heilongjiang province, from June to August in 2021 (Figure 1). After PCR screening, sequencing and BLAST analysis, 35 out of the 80 tick pools were determined as positive for BovHepV. Among the 18 cattle herds, 17 were detected as positive for BovHepV with the positive rate ranging from 0 to 7.8% and the overall positive rate of BovHepV in this study was 10.9% (95% CI: 7.6–14.9%) (Table 2).

**Table 2:** Prevalence of BovHepV detected in ticks collected from different cattle herds in Harbin.

	**H1**	**H2**	**H3**	**H4**	**H5**	**H6**	**H7**	**H8**	**H9**	**H10**	**H11**	**H12**	**H13**	**H14**	**H15**	**H16**	**H17**	**H18**
No. tested	12	15	9	5	14	6	13	14	7	16	8	20	7	9	10	16	14	5
No. positive	3	1	2	1	2	1	4	3	1	2	2	2	1	3	1	4	2	0
Prevalence (%)	5.6	1.4	4.9	4.4	3.0	3.6	7.1	4.7	3.0	2.6	5.6	2.1	3.0	7.8	2.1	5.6	3.0	–

H1–H18 indicates the cattle herd 1 to cattle herd 18.

### 3.2. Sequences comparison of BovHepV

The nearly complete genome sequence of the BovHepV strains amplified from six representative samples showed 99.2–99.7% nucleotide identity and 99.0–100% amino acid identity with each other (Table 3). Furthermore, they shared 67.3–93.2% nucleotide identity and 76.4–98.1% amino acid identity with the BovHepV strains identified world-wide, while they shared the highest identity with the subtype G strains determined from cattle in China (Table 3). Moreover, they shared 81.5–81.8% nucleotide identity and 93.5–94.4% amino acid identity with the subtype H strains (Accession Numbers MZ221927, MZ540979, and MZ540980), which were also identified in blood-sucking tick in China (Table 3).

**Table 3:** The sequence identity within the BovHepV polyprotein at the nucleotide (upper right) and amino acid (lower left, boldface) levels calculated using ClustalW method implemented in MegAlign.

**Strains**	**1**	**2**	**3**	**4**	**5**	**6**	**7**	**8**	**9**	**10**	**11**	**12**	**13**	**14**	**15**	**16**	**17**	**18**	**19**	**20**	**21**	**22**	**23**	**24**	**25**	**26**	**27**	**28**	**29**	**30**	**31**
1		93.8	93.7	93.2	91.5	80.3	79.9	80.2	80.6	80.4	82.9	82.6	79.6	79.6	80.2	84.4	84.1	84.2	84.7	84.7	84.6	84.8	84.8	84.7	84.8	84.9	84.7	81.8	81.7	81.6	66.8
2	**98.2**		93.6	93.3	91.1	80.2	79.9	80.0	80.6	80.4	82.7	82.6	79.7	79.7	80.2	84.5	84.6	84.6	84.8	84.8	84.6	84.8	84.8	84.7	84.8	84.8	84.7	82.1	82.0	82.0	67.0
3	**97.9**	**98.0**		93.5	91.8	80.1	80.1	80.4	80.7	80.5	83.0	82.6	79.8	79.7	80.5	84.5	84.6	84.6	84.4	84.4	84.6	84.8	84.8	84.7	84.7	84.8	84.7	82.0	81.9	81.8	66.9
4	**98.1**	**97.9**	**98.0**		91.6	80.1	79.9	80.1	80.9	80.6	82.6	82.6	79.6	79.7	80.2	84.6	84.6	84.6	84.8	84.8	84.8	85.1	85.0	85.0	85.0	85.1	85.0	81.9	81.8	81.7	67.3
5	**97.4**	**97.5**	**97.4**	**97.5**		79.8	79.9	80.3	80.4	80.3	82.4	82.4	79.1	79.1	79.8	84.4	84.0	84.0	84.3	84.3	84.3	84.4	84.4	84.4	84.5	84.4	84.4	81.9	81.8	81.7	66.9
6	**93.0**	**92.6**	**92.7**	**92.9**	**92.5**		90.4	90.9	82.9	82.8	80.7	80.9	82.1	82.1	81.7	80.4	80.1	80.2	80.2	80.2	80.3	80.3	80.1	80.2	80.3	80.2	80.2	80.2	80.1	80.0	67.3
7	**93.2**	**92.9**	**93.0**	**93.1**	**92.6**	**98.7**		92.1	82.8	82.5	80.7	80.3	81.9	81.8	81.3	80.1	79.9	80.0	80.0	80.0	79.9	79.9	79.8	79.9	80.0	80.0	79.9	79.9	79.8	79.8	67.5
8	**92.9**	**92.8**	**92.9**	**92.8**	**92.6**	**98.6**	**99.0**		83.1	82.8	80.9	80.6	81.9	81.9	81.5	80.4	80.1	80.1	80.0	80.0	80.2	79.9	79.9	80.0	80.0	80.0	79.9	80.2	80.1	80.0	67.3
9	**93.0**	**92.8**	**92.9**	**93.0**	**92.7**	**96.2**	**96.4**	**96.3**		98.4	81.2	81.3	81.8	81.8	81.7	80.1	80.2	80.3	80.8	80.8	80.5	80.5	80.5	80.5	80.7	80.6	80.6	80.0	79.9	79.8	66.8
10	**92.4**	**92.1**	**92.1**	**92.2**	**92.1**	**95.5**	**95.6**	**95.6**	**98.9**		81.0	81.1	81.6	81.6	81.5	79.9	80.1	80.1	80.7	80.7	80.5	80.3	80.2	80.3	80.4	80.4	80.4	79.9	79.7	79.7	66.9
11	**94.3**	**93.6**	**93.7**	**94.0**	**93.8**	**93.2**	**93.2**	**93.0**	**93.0**	**92.4**		93.9	80.9	80.8	80.2	82.6	82.5	82.5	82.6	82.6	82.6	82.5	82.5	82.6	82.6	82.6	82.5	84.8	84.7	84.6	67.2
12	**94.5**	**93.9**	**93.9**	**94.1**	**93.9**	**93.2**	**93.1**	**93.0**	**93.2**	**92.6**	**98.1**		80.3	80.4	80.1	82.3	82.2	82.2	82.1	82.1	82.4	82.3	82.3	82.4	82.4	82.4	82.3	84.6	84.5	84.4	66.9
13	**92.0**	**91.9**	**91.9**	**91.8**	**91.4**	**95.4**	**95.2**	**95.3**	**94.8**	**94.4**	**92.3**	**92.4**		99.8	81.2	80.0	79.4	79.4	79.5	79.5	79.4	79.4	79.3	79.4	79.4	79.4	79.3	80.7	80.5	80.5	67.1
14	**92.0**	**91.9**	**91.9**	**91.9**	**91.4**	**95.4**	**95.3**	**95.4**	**94.8**	**94.4**	**92.4**	**92.5**	**100**		81.1	80.0	79.4	79.4	79.4	79.4	79.3	79.4	79.3	79.3	79.4	79.4	79.3	80.7	80.5	80.4	67.1
15	**92.1**	**91.9**	**92.1**	**92.0**	**91.9**	**95.1**	**94.9**	**94.9**	**95.0**	**94.4**	**92.7**	**92.5**	**94.3**	**94.3**		79.6	79.9	79.9	80.0	80.0	79.7	79.6	79.5	79.6	79.5	79.5	79.5	79.8	79.6	79.6	67.5
16	**95.7**	**95.4**	**95.7**	**95.6**	**95.6**	**93.1**	**93.2**	**93.0**	**93.0**	**92.5**	**94.8**	**94.7**	**92.2**	**92.2**	**92.3**		92.8	92.8	92.0	92.0	92.3	93.0	92.8	92.9	93.2	93.1	93.2	81.7	81.6	81.5	67.6
17	**95.5**	**95.3**	**95.6**	**95.5**	**95.4**	**92.6**	**92.8**	**92.6**	**92.7**	**92.2**	**94.4**	**94.5**	**92.0**	**92.0**	**92.0**	**97.9**		99.9	91.5	91.5	92.5	92.7	92.7	92.8	92.7	92.8	92.6	81.4	81.3	81.2	67.3
18	**95.5**	**95.3**	**95.6**	**95.5**	**95.4**	**92.6**	**92.8**	**92.6**	**92.7**	**92.2**	**94.4**	**94.5**	**92.0**	**92.0**	**92.0**	**97.9**	**100**		91.6	91.6	92.5	92.8	92.7	92.8	92.8	92.8	92.7	81.4	81.3	81.2	67.4
19	**96.1**	**95.6**	**96.0**	**96.0**	**95.9**	**93.4**	**93.6**	**93.3**	**93.3**	**92.7**	**95.0**	**94.9**	**92.2**	**92.2**	**92.6**	**98.0**	**98.0**	**98.0**		100	92.2	91.6	91.5	91.6	91.7	91.7	91.5	81.9	81.8	81.7	67.4
20	**96.1**	**95.6**	**96.0**	**96.0**	**95.9**	**93.4**	**93.6**	**93.3**	**93.3**	**92.7**	**95.0**	**94.9**	**92.2**	**92.2**	**92.6**	**98.0**	**98.0**	**98.0**	**100**		92.2	91.6	91.5	91.6	91.7	91.7	91.5	81.9	81.8	81.7	67.4
21	**95.9**	**95.4**	**95.8**	**95.8**	**95.7**	**92.7**	**93.0**	**92.7**	**92.6**	**92.1**	**94.5**	**94.4**	**91.9**	**91.9**	**91.8**	**97.7**	**97.6**	**97.6**	**98.0**	**98.0**		92.6	92.5	92.6	92.6	92.6	92.6	81.9	81.8	81.7	67.5
22	**95.7**	**95.3**	**95.7**	**95.6**	**95.4**	**92.7**	**93.0**	**92.7**	**92.7**	**92.1**	**94.6**	**94.7**	**91.9**	**91.9**	**91.9**	**97.8**	**97.7**	**97.7**	**97.9**	**97.9**	**97.5**		99.4	99.5	99.3	99.3	99.2	81.8	81.7	81.6	67.4
23	**95.6**	**95.2**	**95.6**	**95.5**	**95.3**	**92.6**	**92.8**	**92.5**	**92.6**	**92.0**	**94.6**	**94.6**	**91.8**	**91.8**	**91.7**	**97.6**	**97.6**	**97.6**	**97.7**	**97.7**	**97.3**	**99.4**		99.7	99.3	99.5	99.2	81.7	81.6	81.5	67.4
24	**95.7**	**95.4**	**95.7**	**95.6**	**95.5**	**92.8**	**93.0**	**92.7**	**92.8**	**92.2**	**94.7**	**94.8**	**92.0**	**92.0**	**91.9**	**97.7**	**97.7**	**97.7**	**97.9**	**97.9**	**97.4**	**99.5**	**99.7**		99.5	99.3	99.2	81.8	81.6	81.6	67.5
25	**95.7**	**95.3**	**95.7**	**95.6**	**95.5**	**92.8**	**93.0**	**92.7**	**92.8**	**92.2**	**94.8**	**94.8**	**91.9**	**91.9**	**91.9**	**98.0**	**97.7**	**97.7**	**97.8**	**97.8**	**97.5**	**99.2**	**99.2**	**99.5**		99.5	99.4	81.8	81.6	81.6	67.4
26	**95.7**	**95.3**	**95.7**	**95.6**	**95.5**	**92.8**	**93.0**	**92.7**	**92.8**	**92.2**	**94.8**	**94.8**	**91.9**	**91.9**	**91.9**	**98.0**	**97.7**	**97.7**	**97.8**	**97.8**	**97.5**	**99.2**	**99.2**	**99.5**	**100**		99.4	81.8	81.7	81.6	67.3
27	**95.6**	**95.2**	**95.6**	**95.5**	**95.4**	**92.7**	**92.9**	**92.6**	**92.7**	**92.1**	**94.6**	**94.6**	**91.9**	**91.9**	**91.7**	**98.1**	**97.5**	**97.5**	**97.7**	**97.7**	**97.5**	**99.1**	**99.0**	**99.1**	**99.4**	**99.4**		81.8	81.6	81.6	67.3
28	**94.4**	**94.1**	**93.9**	**94.1**	**94.0**	**92.6**	**92.7**	**92.6**	**92.6**	**92.0**	**95.5**	**95.6**	**91.7**	**91.8**	**92.0**	**94.6**	**94.2**	**94.2**	**94.6**	**94.6**	**94.3**	**94.4**	**94.2**	**94.4**	**94.4**	**94.4**	**94.3**		99.7	99.6	66.8
29	**94.0**	**93.8**	**93.5**	**93.7**	**93.6**	**92.1**	**92.2**	**92.2**	**92.1**	**91.6**	**95.0**	**95.1**	**91.3**	**91.4**	**91.5**	**94.1**	**93.7**	**93.7**	**94.2**	**94.2**	**93.9**	**93.9**	**93.8**	**94.0**	**94.0**	**94.0**	**93.8**	**94.3**		99.8	66.8
30	**93.8**	**93.6**	**93.2**	**93.4**	**93.4**	**91.9**	**92.0**	**92.0**	**91.9**	**91.4**	**94.8**	**94.9**	**91.1**	**91.1**	**91.3**	**93.9**	**93.5**	**93.5**	**93.9**	**93.9**	**93.6**	**93.6**	**93.5**	**93.7**	**93.7**	**93.7**	**93.6**	**93.8**	**99.7**		66.7
31	**76.4**	**76.4**	**76.1**	**76.3**	**76.3**	**76.2**	**76.4**	**76.3**	**76.7**	**76.6**	**76.6**	**76.6**	**75.9**	**75.9**	**76.5**	**76.7**	**76.3**	**76.3**	**76.6**	**76.6**	**76.6**	**76.5**	**76.4**	**76.6**	**76.6**	**76.6**	**76.5**	**93.6**	**76.3**	**76.1**	

The compared strains were indicated as accession number and country, and the different colors mean the different subtypes/genotypes of the BovHepV strains.

GenBank accession number: 1 (KP641125); 2 (KP641123); 3 (KP641127); 4 (KP641124); 5 (KP641126); 6 (KP265950); 7 (KP265948); 8 (KP265943); 9 (KP265947); 10 (KP265946); 11 (MG781019); 12 (MG781018); 13 (MG257793); 14 (MG257794); 15 (MH027948); 16 (MN266283); 17 (MN266285); 18 (MN266284); 19 (OM131409); 20 (OM131410); 21 (MW830376); 22 (OP716808); 23 (OP716809); 24 (OP716810); 25 (OP716811); 26 (OP716812); 27 (OP716813); 28 (MZ221927); 29 (MZ540979); 30 (MZ540980); 31 (MN691105).

### 3.3. Recombination and phylogenetic analysis of BovHepV

**Figure 2:**
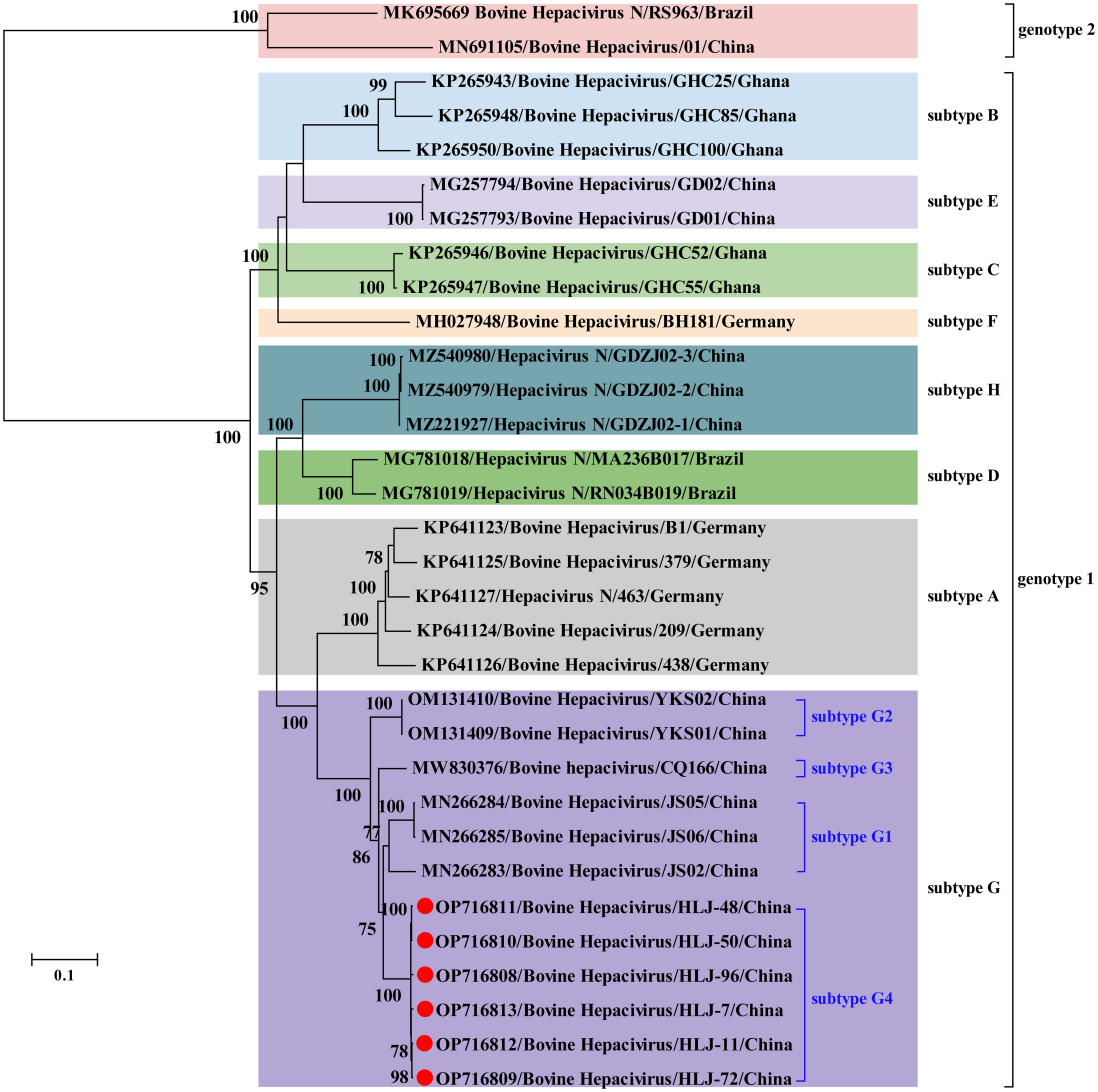
Phylogenetic analysis based on the nucleotide sequences of complete polyprotein-coding region of BovHepV including the newly identified sequences and other reference sequences retrieved from GenBank. The trees were constructed based on the maximum likelihood method implemented in MEGA 7.0, and mid-point rooted for clarity and the scale bar represents the number of nucleotide substitutions per site. Bootstrap values were calculated with 100 replicates of the alignment, and only bootstrap values > 70% are shown at relevant nodes. GenBank accession numbers are followed by the name of hepacivirus strains. Red dots indicate the BovHepV determined in this study.

No statistically supported recombination event was detected within BovHepV strains after systematic analyzes. Phylogenetic analysis reconstructed based on the complete polyprotein coding sequence showed that all BovHepV strains were divided into two genotypes (genotype 1 and genotype 2), and genotype 1 strains are clearly divided into eight well-separated subtypes (subtype A–G). The six viruses identified in this study are more closely related to subtype G viruses that identified in bovine samples collected in Jiangsu, Chongqing, and Inner Mongolia, China, while was distinguished from the subtype H viruses identified in ticks in Guangdong province, China. Notably, virus strains identified in this study showed a closer phylogenetic relationship with those viruses identified in Jiangsu than the Inner Mongolia ones (OM131409–OM131410), although Inner Mongolia is geographically closer to Heilongjiang province (Figure 2).

## 4. Discussion

Since its first discovery in cattle in Germany and Ghana in 2015, BovHepV has been identified in seven continents (21, 22, 24, 27–30), indicating the worldwide geographical distribution of BovHepV. Moreover, the currently identified BovHepV could be classified into two genotypes, and genotype 1 could be further divided into eight subtypes, suggesting the extensive genetic diversity of BovHepV (24). Interestingly, in this study, the viral RNA of BovHepV was detected in blood-sucking ticks, suggesting that ticks may be serve as an arthropod vector for hepacivirus. However, no information about bovine hepacivirus in ticks collected from vegetation. Moreover, although bovine hepacivirus have been detected in blood-sucking ticks in this study and our previous study (24), we did not simultaneously analyze the active infection in the cattle that the questing tick removed. Together, these results suggest that ticks may be a transmission vector of BovHepV, although it needs further investigation.

In China, BovHepV has been detected in Guangdong, Jiangsu, Yunnan, Sichuan, Heilongjiang, Shandong, Henan, Inner Mongolia and Chongqing (25, 31–34), suggesting that BovHepV was circulating in cattle herds in a wide geographic in China. Notably, BovHepV strains identified in China were segregated into two genotypes and thee subtypes in subtype 1 in the phylogenetic tree. In addition, previous study performed in Inner Mongolia further divided the subtype G into subtype G1–G2 (31). In this study, BovHepV strains in subtype G were divided into four clades (subtype G1–G4), and the BovHepV strains identified in this study were classified as the subtype G4. These results imply the extensive genetic diversity of BovHepV that circulates in China. Additionally, among all genotypes or subtypes of BovHepV identified in China, the viruses belonging to the subtype G were widely detected in Jiangsu, Chongqing, Inner Mongolia, and Heilongjiang, China, indicating that BovHepV in subtype G may be the primary circulating subtype in China.

Previous studies have shown that BovHepV subtypes are associated with their geographic origins based on the limited number of BovHepV sequences (21, 22, 26, 28), and our results were generally consistent with this conclusion. However, the results in this study also show the complex geographic distribution of BovHepV genotypes or subtypes. For example, viruses in subtypes E, H, and G that were identified in China showed a closer phylogenetic relationship with those in subtypes B, D, and A, which were identified in Ghana, Brazil, and Germany, respectively. This could have resulted from frequent international trade of live cattle, which can facilitate transboundary transmission of BovHepV. In addition, the BovHepV strains detected in this study showed a closer phylogenetic relationship with those identified in Jiangsu than the Inner Mongolia strains, although Inner Mongolia is geographically closer to Heilongjiang province. These results indicating an intriguing evolutionary route of BovHepV.

In conclusion, BovHepV belong to the subtype G was detected in *Rhipicephalus microplus* ticks collected from cattle in Heilongjiang province, northeastern China with an overall prevalence of 10.9%. This is the first reports about the detection of BovHepV in ticks in Heilongjiang province, which expands our knowledge that ticks may be a transmission vector of BovHepV.

## Data availability statement

The datasets presented in this study can be found in online repositories. The names of the repository/repositories and accession number(s) can be found below: https://www.ncbi.nlm.nih.gov/genbank/, OP716808–OP716813.

## Ethics statement

The animal study was reviewed and approved by the Ethics Committee of College of Life Science and Engineering, Foshan University. Written informed consent was obtained from the owners for the participation of their animals in this study.

## Author contributions

X-LZ and J-WS: conceived, designed the experiments, and writing—review and editing. SY, X-YY, C-YL, and SK: collect the samples, performed the experiments, and analyzed the data. SK: help to collect the samples. X-YY: writing—original draft preparation. All authors contributed to the article and approved the submitted version.
